# The prevalence of developmental coordination disorder in children: a systematic review and meta-analysis

**DOI:** 10.3389/fped.2024.1387406

**Published:** 2024-09-26

**Authors:** Huaqiang Li, Xiaohua Ke, Dunbing Huang, Xiaqing Xu, Huan Tian, Jiaxin Gao, Cai Jiang, Wei Song

**Affiliations:** ^1^ School of Health Preservation and Rehabilitation, Chengdu University of Traditional Chinese Medicine, Chengdu, China; ^2^ Department of Rehabilitation Medicine, Shanghai Fourth People’s Hospital, School of Medicine, Tongji University, Shanghai, China; ^3^ Shengli Clinical Medical College of Fujian Medical University, Fuzhou, China; ^4^ Rehabilitation Department, Fujian Provincial Hospital, Fuzhou, China

**Keywords:** child, developmental coordination disorder, prevalence, meta-analysis, review

## Abstract

**Purpose:**

The aim of the study was to synthesize previous evidence and clarify the prevalence of developmental coordination disorder (DCD) in children by meta-analysis.

**Methods:**

A comprehensive computerized search of databases, including PubMed, Embase, Web of Science, The Cochrane Library, CINAHL, and PsycINFO databases, was conducted to identify relevant national and international articles published before 18 December 2023 on DCD prevalence in children. The meta-analysis of prevalence was conducted using Stata 18.0.

**Results:**

A total of 18 papers involving 31,203 patients were included. The prevalence of children with DCD was found to be 5%. A subgroup analysis showed that prevalence was 7% [95% confidence interval (CI) 4%–10%] and 4% (95% CI 3%–7%) for boys and girls, respectively; 4% (95% CI 2%–8%), 2% (95% CI 2%–2%), and 6% (95% CI 3%–10%) in Asia, Europe, and North America, respectively; and 18% (95% CI 8%–31%) and 6% (95% CI 4%–7%) for preterm (<37 weeks) and term infants (≥37 weeks), respectively. The prevalence of very low birth weight children (<1,250 g) with DCD was found to be 31%.

**Conclusion:**

In this study, we found that the prevalence of children with DCD in the general population was 5% and that preterm infants (<37 weeks) and very low birth weight infants (<1,250 g) have a higher prevalence of DCD and require early screening and regular follow-up.

**Systematic Review Registration:**

https://www.crd.york.ac.uk/, Identifier (CRD42024503320).

## 1 Introduction

Developmental coordination disorder (DCD) is a neurodevelopmental disorder characterized by a significantly impaired ability to learn and perform coordinated motor skills (1). Children diagnosed with DCD exhibit significant brain differences in areas of the cerebellum, basal ganglia, corpus callosum, parietal lobes, and frontal lobes compared with normal children, and these neurological differences continue to affect the functional development of adolescents (2). Children with DCD show negative effects on academic productivity, physical education, and activities of daily living (dressing, brushing teeth, etc.) (3, 4), Timely attention and intervention by parents, teachers, and the community are needed. Without timely intervention, motor skill deficits associated with DCD may persist into adolescence and adulthood (5, 6). Adults with DCD have persistent difficulties in a range of motor skills and in learning new skills (driving, musical instruments), as well as lower physical endurance, flexibility, and strength, and poorer overall health (mental and physical) than adults without DCD (7–9).

The motor difficulties of people with DCD are often considered “mild” compared to those with severe movement disorders, such as cerebral palsy; therefore, it may be assumed that DCD is not a cause for concern. However, studies have found significant effects of DCD on daily activities and academic performance, followed by significant effects on social participation, physical health, and mental health problems, which, together with the high prevalence of the condition, suggests that the social and economic burden is considerable (10). In recent years, research on the pathogenesis of DCD has grown almost exponentially but remains inconclusive (10). The more recognized effects are as follows: (1) under-activation of functional networks: it has been found that young children with DCD have reduced activation in the parietal lobe, cerebellar axis, and posterior cerebellar regions, leading to abnormalities in motor planning, motor control, visual-motor mapping, and automatisms, which affect children's motor control and learning function. Recent imaging results have also shown that both structural and functional neural activation patterns are disrupted in children with DCD (11, 12); (2) internal modeling deficit (IMD) hypothesis: internal modeling deficits affect a young child's ability to make motor adjustments in response to changes in the external environment, impairing motor automatisms and learning function. In addition, disruption of the mirror neuron system (MNS) and delayed maturation of atypical interhemispheric communication has been found to be associated with DCD (10, 13). The pathogenesis of DCD should be further explored in the future.

The 2019 International Clinical Guidelines state that the prevalence of DCD in children is currently estimated to be in the range of 2%–20%, with 5%–6% often cited in the literature (10). Currently, the prevalence of DCD reported in studies from different countries varies widely, ranging from a high of 13.4% (14) to a low of 0.8% (15). Incorrect prevalence rates can influence medical decisions, allocation of healthcare resources, and direction of medical research. However, some previous studies have been limited to specific populations (preterm births, obesity, etc.) or have used motor function tests alone. Movement Assessment Battery for Children (MABC) (16) or DCD questionnaires alone (17, 18), ignoring other criteria to diagnose DCD, will undoubtedly lead to a biased prevalence rate. Therefore, only studies diagnosed with DCD based on Diagnostic and Statistical Manual of Mental Disorders IV/V (DSM4/5) criteria recommended in the 2019 International DCD Clinical Guidelines were included in our analysis. In addition, to obtain the prevalence of DCD in children in general, we excluded studies with limited study populations (preterm, obese, etc.) from the overall prevalence analysis and only performed subgroup analyses. The aim of the study was to provide strong evidence on the prevalence of DCD to justify early clinical screening and preventive measures for DCD.

## 2 Methods

### 2.1 Agreements and registrations

The Systematic Evaluation Program has been registered in the PROSPERO international database and accepted on 27 January 2024 (registration no. CRD42024503320).

### 2.2 Inclusion and exclusion criteria

#### 2.2.1 Inclusion criteria

1. Children aged 3–17 years.
2. Study design: observational studies, including cohort studies, case–control studies, cross-sectional studies, etc.
3. The diagnosis of DCD according to the DSM4/5 (10) fulfilled the following four criteria: the acquisition and execution of coordinated motor skills is substantially below that expected given the individual's chronological age and sufficient opportunities to acquire age-appropriate motor skills; the motor skills significantly and persistently interfere with the activities of everyday living appropriate to chronological age and impact upon academic/school productivity, prevocational and vocational activities, leisure, and play; the motor skills deficits are not better accounted for by any other medical, neurodevelopmental, psychological, social condition, or cultural background; and onset of symptoms in childhood.

#### 2.2.2 Exclusion criteria

1. Duplicate publications or literature with the same original data.
2. Incomplete or unavailable analyses of relevant data.

### 2.3 Search strategy

The Preferred Reporting Items for Systematic Reviews and Meta-Analyses (PRISMA) guidelines were used to construct this review (19). The following databases were searched on 18 December 2023: PubMed, The Cochrane Library, Embase, Web of Since, CINAHL, and PsycINFO. Two researchers (HL and XK) independently reviewed the titles, abstracts, and full text of the search results to locate included studies. Two researchers reviewed conflicts and consulted a third independent reviewer (WS) if a decision could not be made. The Reference lists of the included studies were screened to identify any missing studies not found in the initial search that met the inclusion criteria. Supplementary Material Data Sheet 1 summarizes the search strategy used.

### 2.4 Literature screening and data extraction

Two researchers (HL, DH) independently screened the literature and extracted information to ensure correct data. First, titles and abstracts were read after excluding duplicate titles and initial screening was carried out based on inclusion and exclusion criteria. Literature that was uncertain during the initial screening process and required further assessment was read in full and then studies were identified for inclusion based on the inclusion and exclusion criteria. Disagreements arising from the literature screening and data extraction process were discussed with the third senior specialist (WS) and decided. A data extraction sheet was created through Microsoft Excel to record basic information about the study—including the first author's name, age of children, country, and sample size—and outcome indicators: the number of sick children. Finally, the literature and information were cross-checked, and for studies with missing data, the corresponding authors of the literature were contacted and added on time.

### 2.5 Statistical analysis

Data from the included literature were statistically analyzed using Stata 18.0 software. The prevalence of DCD was used as the effect size and the 95% confidence interval (CI) for the prevalence was calculated. Heterogeneity was tested using *I*^2^; if *p* > 0.1 and *I*^2^ < 50%, the heterogeneity between studies was considered small and the effect sizes were combined using a fixed-effects model; if *p* ≤ 0.1 and *I*^2^ ≥ 50%, the heterogeneity between studies was considered significant and the effect sizes were combined using a random-effects model, and sensitivity and subgroup analyses were used to find the sources of heterogeneity further. Two funnel plots, Begg's test and Egger's test, were used to analyze the presence of potential publication bias. Differences were considered statistically significant at *p* < 0.05.

### 2.6 Quality assessment

The included literature was independently quality assessed by two researchers (HL, DH) and disagreements during the assessment process were resolved through discussion with the senior specialist (WS). Cross-sectional studies were evaluated using the Agency for Healthcare Research and Quality (AHRQ) instrument, which consists of 11 entries, with a score of 1 for “yes” and 0 for “no” or “unclear.” The total score was 11 points, with 8–11 points for high quality, 4–7 points for medium quality, and 0–3 points for low quality. Cohort studies and case–control studies were evaluated for quality using the Newcastle-Ottawa Scale for cohort studies, which consists of eight entries with a total score of 9 points, mainly including the selection of the study population (0–4 points), comparability between groups (0–2 points), and the measurement of exposure factors (0–3 points). Scores of 7–9 points were considered to be of high quality, 5–6 points were considered to be of moderate quality, and 0–4 points were considered to be of low quality.

## 3 Results

### 3.1 Literature search results

Relevant literature was obtained through database searches (*n* = 4,579) as follows: PubMed (*n* = 730), Web of Science (*n* = 1,792), Embase (*n* = 736), CINAHL (*n* = 545), PsycINFO (*n* = 235), and The Cochrane Library (*n* = 541). The initial search yielded 4,579 potentially eligible studies; however, 879 duplicates were removed manually and using EndNote software, 2,912 subject inconsistencies were removed after reading the titles and abstracts, 725 studies had design inconsistencies, 33 studies were removed after reading the full text, and 12 studies had a DCD diagnosis that did not meet DSM4/5 criteria. Finally, 18 studies were eligible for inclusion. Of them, three studies were conducted in China (20–22), five were from Canada (23–27), two were from India (15, 28), and the remaining studies were from Brazil (*n* = 1) (29), Sweden (*n* = 2) (14, 30), the United Kingdom (*n* = 1) (31), Australia (*n* = 1) (32), Finland (*n* = 1) (33), Korea (*n* = 1) (34), and Italy (*n* = 1) (35).

**Figure 1:**
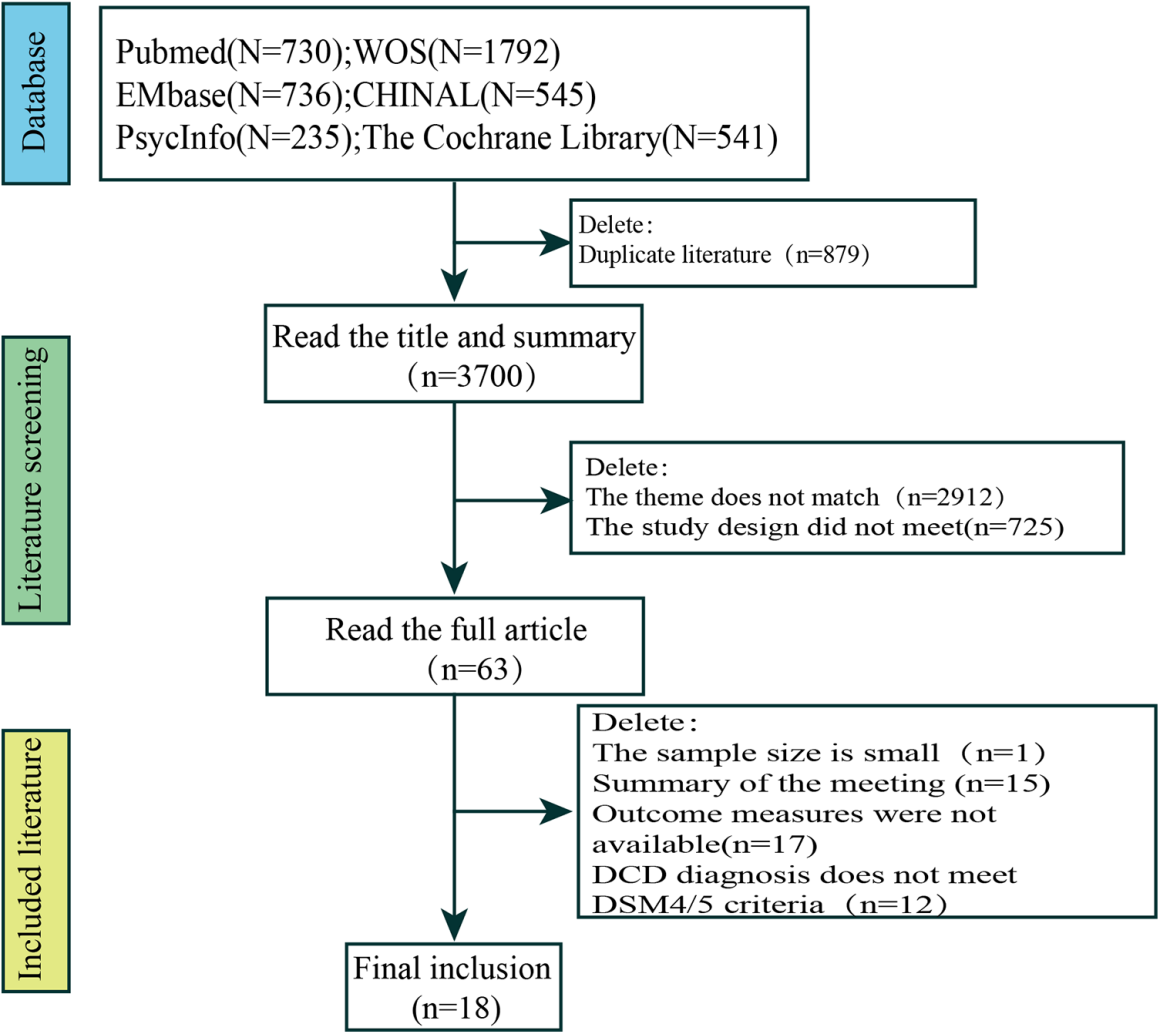
Literature screening flowchart.

A total of 18 studies were included in the final analysis. The search process and exclusion of the stages of the main reasons for study exclusion are shown in Figure 1. The basic characteristics of the included studies are shown in Table 1.

**Table 1:** Characteristics of included studies.

Author	Year	Country	Age	Sample	DCD	Study design	Quality evaluation
Li et al. (20)	2023	China	4–12	825	80	Cross-section	6
Sujatha et al. (28)	2020	India	8–17	944	36	Cross-section	8
Uusitalo et al. (33)	2020	Finland	11	160	18	Cohort	6
Yang et al. (21)	2020	China	3–6	8,586	571	Cross-section	5
Lee et al. (34)	2019	Korea	8–9	548	6	Cross-section	9
Caravale et al. (35)	2019	Italy	8–11	608	75	Cohort	8
Bolk et al. (30)	2018	Sweden	6.5	229	85	Cohort	7
Girish et al. (15)	2016	India	6–15	2,263	19	Cross-section	5
Cardoso et al. (29)	2014	Brazil	7–8	793	34	Cross-section	6
Hua et al. (22)	2014	China	3–6	4,001	330	Cross-section	6
Rivard et al. (23)	2014	Canada	8–15	3,070	122	Cross-section	5
Zwicker et al. (24)	2013	Canada	4–5	157	45	Cohort	6
Roberts et al. (32)	2011	Australia	8	132	21	Cohort	5
Lingam et al. (31)	2009	United Kingdom	7	6,990	123	Cohort	8
Cairney et al. (25)	2005	Canada	9–14	578	44	Cross-section	6
Hay et al. (26)	2004	Canada	11.5	206	17	Cross-section	6
Holsti et al. (27)	2002	Canada	9	73	37	Cohort	5
Kadesjö and Gillberg (14)	1999	Sweden	7	409	55	Cross-section	7

### 3.2 Basic characteristics and quality assessment of the included literature

A total of 31,203 children (age range 3–17 years) were included in the analysis. All studies confirmed the diagnosis of DCD based on DSM4/5 diagnostic guidelines. The results are shown in Supplementary Table 1.

### 3.3 Meta-analysis results

#### 3.3.1 Prevalence of DCD

**Figure 2:**
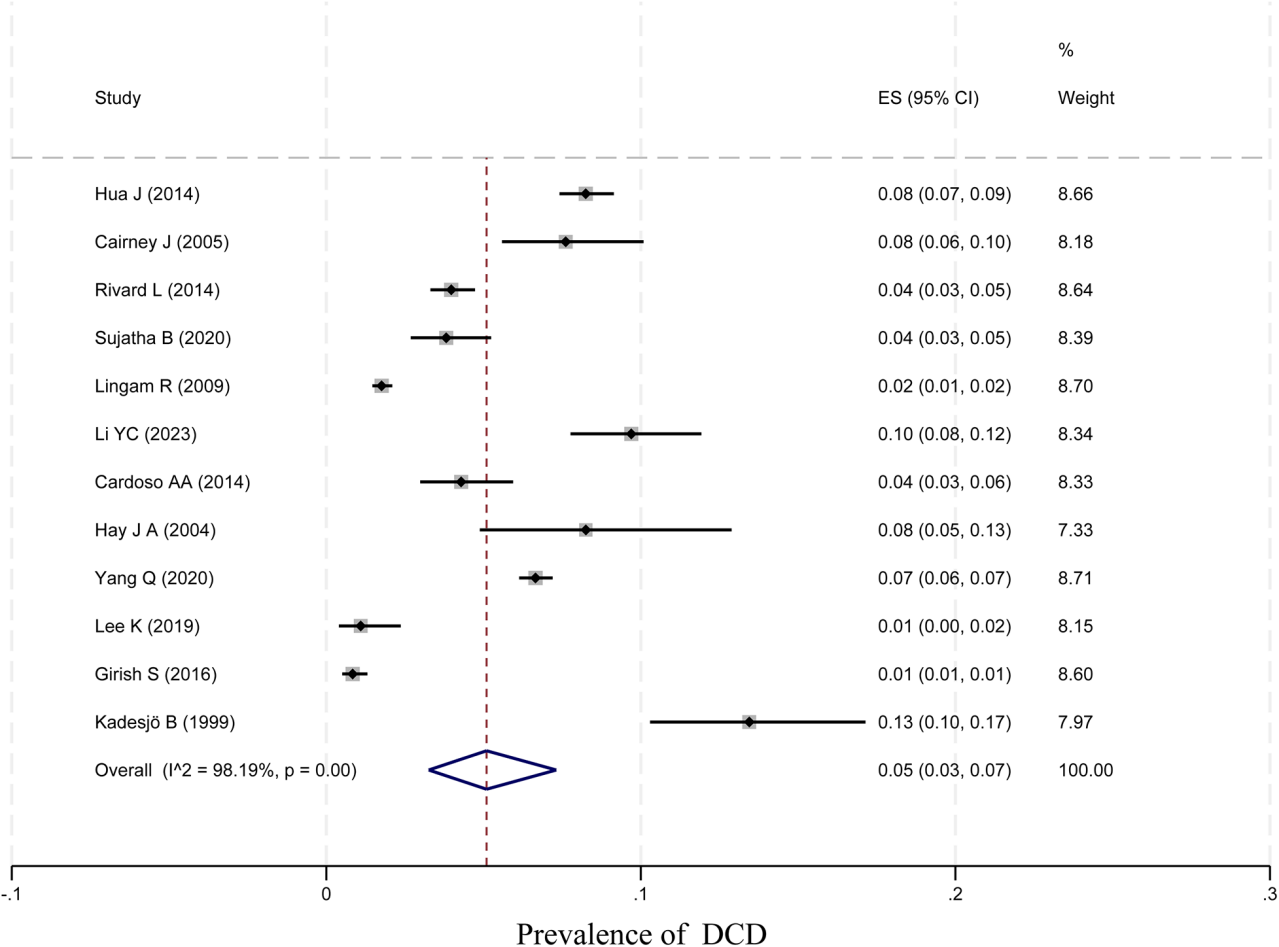
Forest map.

In total, 12 papers were tested for heterogeneity, which showed significant heterogeneity, and a meta-analysis was performed using a random-effects model. The results showed that the prevalence of DCD among children was 5% (95% CI 3%–7%; *I*^2^ = 98.19, *p* < 0.01, 29,213 children). The specific results are shown in Figure 2.

#### 3.3.2 Subgroup analysis

**Figure 3:**
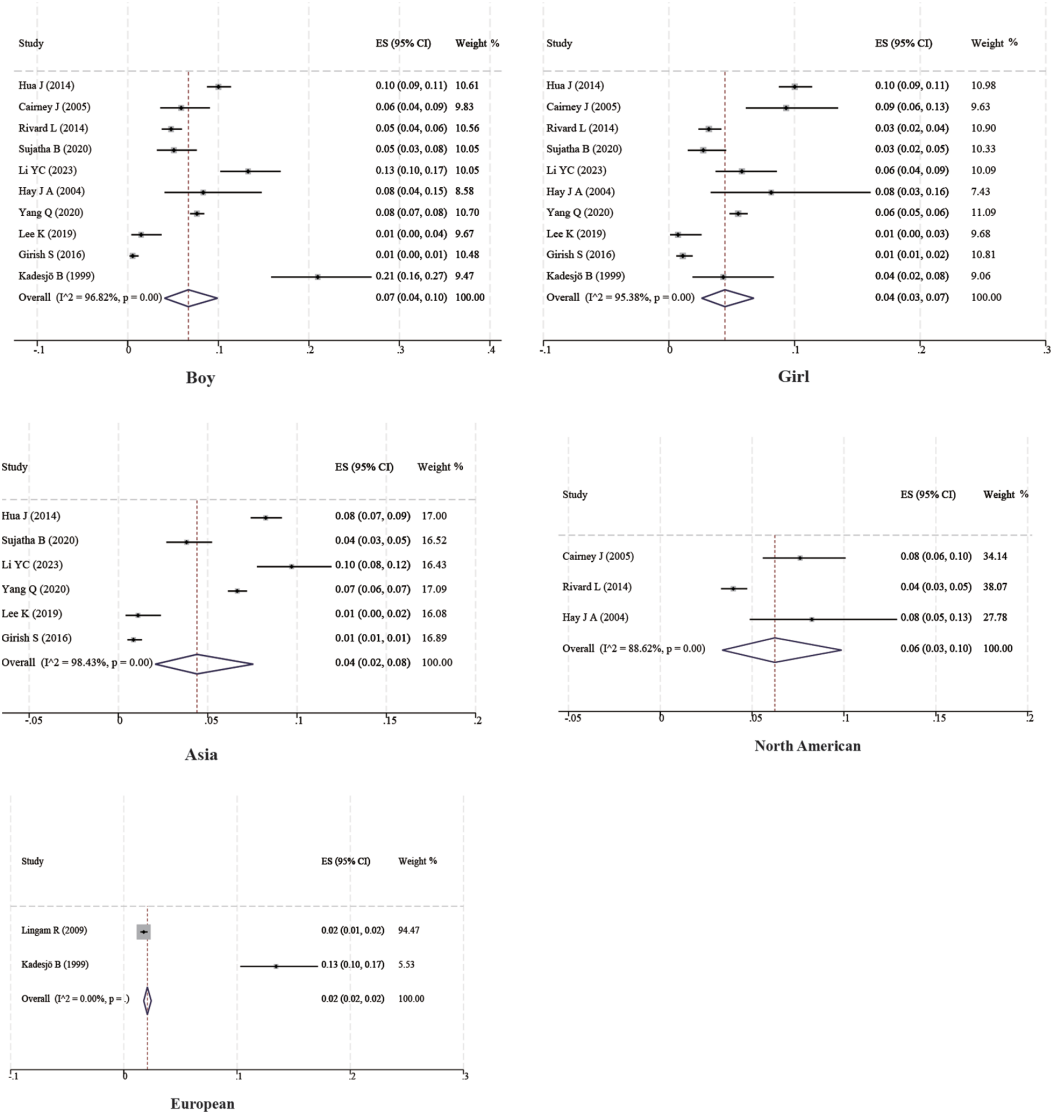
Subgroup forest map.

**Figure 4:**
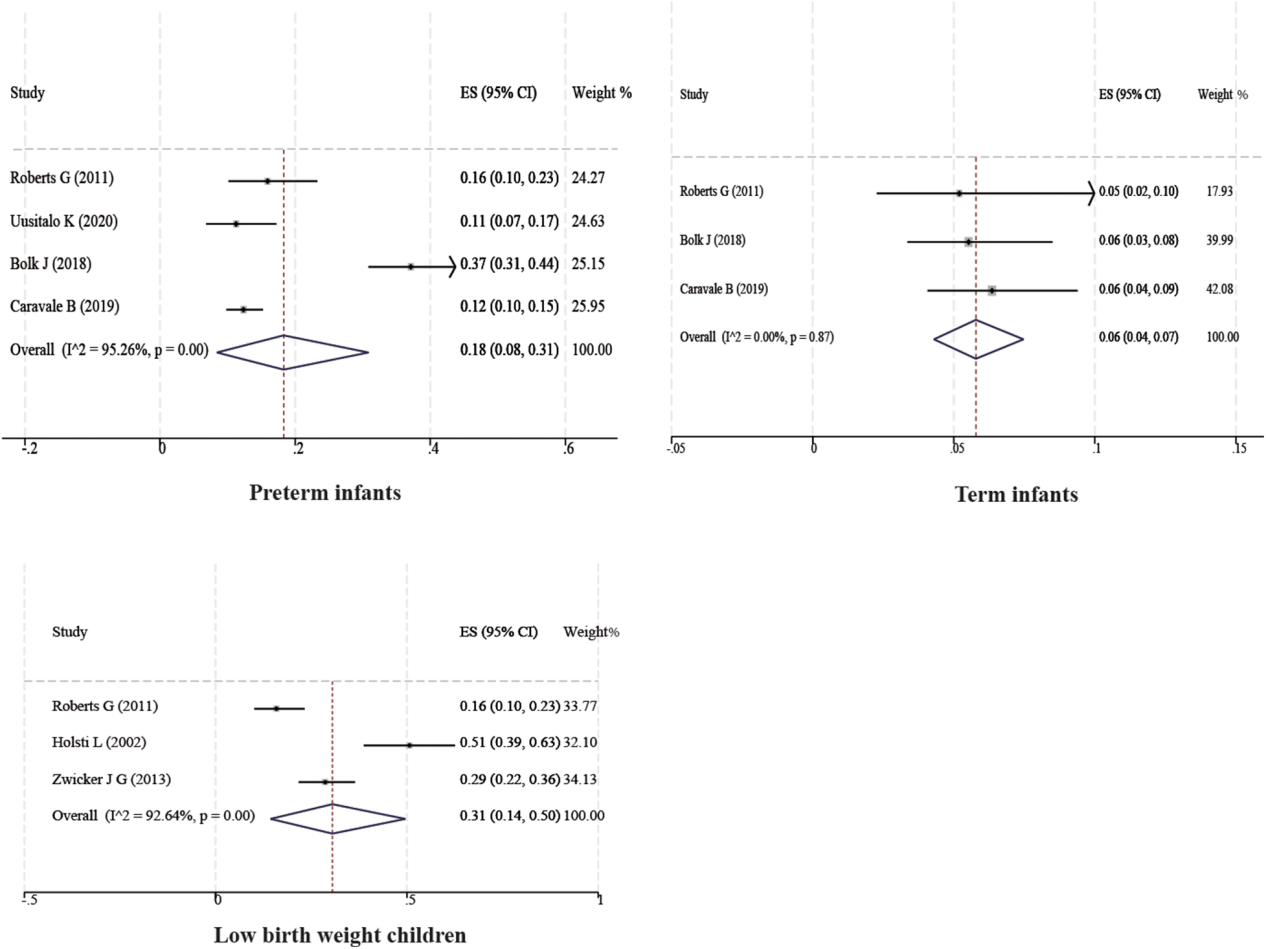
Subgroup forest map.

Ten studies with a total of 11,090 boys and 10,504 girls were included to characterize the prevalence of DCD between sexes, and the prevalence was found to be 7% (95% CI 4%–10%) and 4% (95% CI 3%–7%) for boys and girls, respectively; six studies with a total of 17,167 children were included to characterize the prevalence of DCD in Asian children, and the prevalence was found to be 4% (95% CI 2%–8%). Two studies with a total of 7,399 children were included to characterize the prevalence of DCD in European children, and the prevalence was found to be 2% (95% CI 2%–2%). Three studies with a total of 3,854 children were included to characterize the prevalence of DCD in North American children, and the prevalence was found to be 6% (95% CI 3%–10%). In addition, four studies with 1,129 children were included to characterize the prevalence of DCD in preterm infants (<37 weeks), and the prevalence of DCD was found to be 18% (95% CI 8%–31%). Three studies with 860 children were included to characterize the prevalence of DCD in term infants (≥37 weeks), and the prevalence of DCD was found to be 6% (95% CI 4%–7%). Three studies with 362 children were included to characterize the prevalence of DCD in very low birth weight children (VLBWI) (<1,250 g), and the prevalence of DCD in low birth weight children was found to be 6% (95% CI 4%–10%). Three studies with a total of 362 children were included to analyze the prevalence of DCD in very low birth weight children (<1,250 g), and the prevalence of DCD in very low birth weight children was found to be 31% (95% CI 14%–50%). The results are shown in Table 2 and Figures 3, 4.

**Table 2:** Results of subgroup analysis.

Classification	Inclusion of literature	Sample size (children)	Heterogeneous results		Consolidation model	Metal results	95% CI
			*I* ^2^	*P*			
Genders							
Male (14, 15, 20–23, 25, 26, 28, 34)	10	11,090	96.82	<0.001	Random	7	4–10
Female (14, 15, 20–23, 25, 26, 28, 34)	10	10,504	95.38	<0.001	Random	4	3–7
Area							
Asia (15, 20–22, 28, 34)	6	17,167	98.43	<0.001	Random	4	2–8
Europe (14, 31)	2	7,399	—	—	Random	2	2–2
North America (23, 25, 26)	3	3,854	88.62	<0.001	Random	6	3–10
Delivery							
Premature baby (30, 32, 33, 35)	4	1,129	95.26	<0.001	Random	18	8–31
Term baby (21, 30, 35)	3	860	0	0.85	Fix	6	4–7
Low birth weight children (24, 27, 32)	3	362	92.64	<0.001	Random	31	14–50

#### 3.3.3 Sensitivity analysis

**Figure 5:**
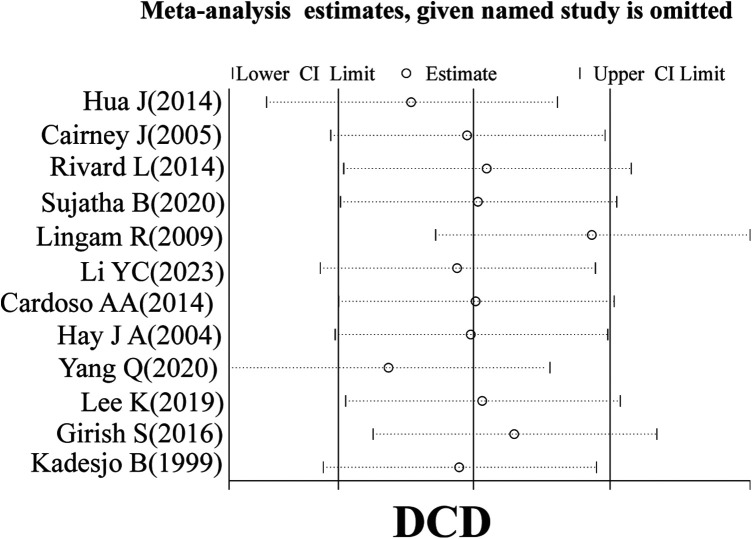
Sensitivity analysis.

**Figure 6:**
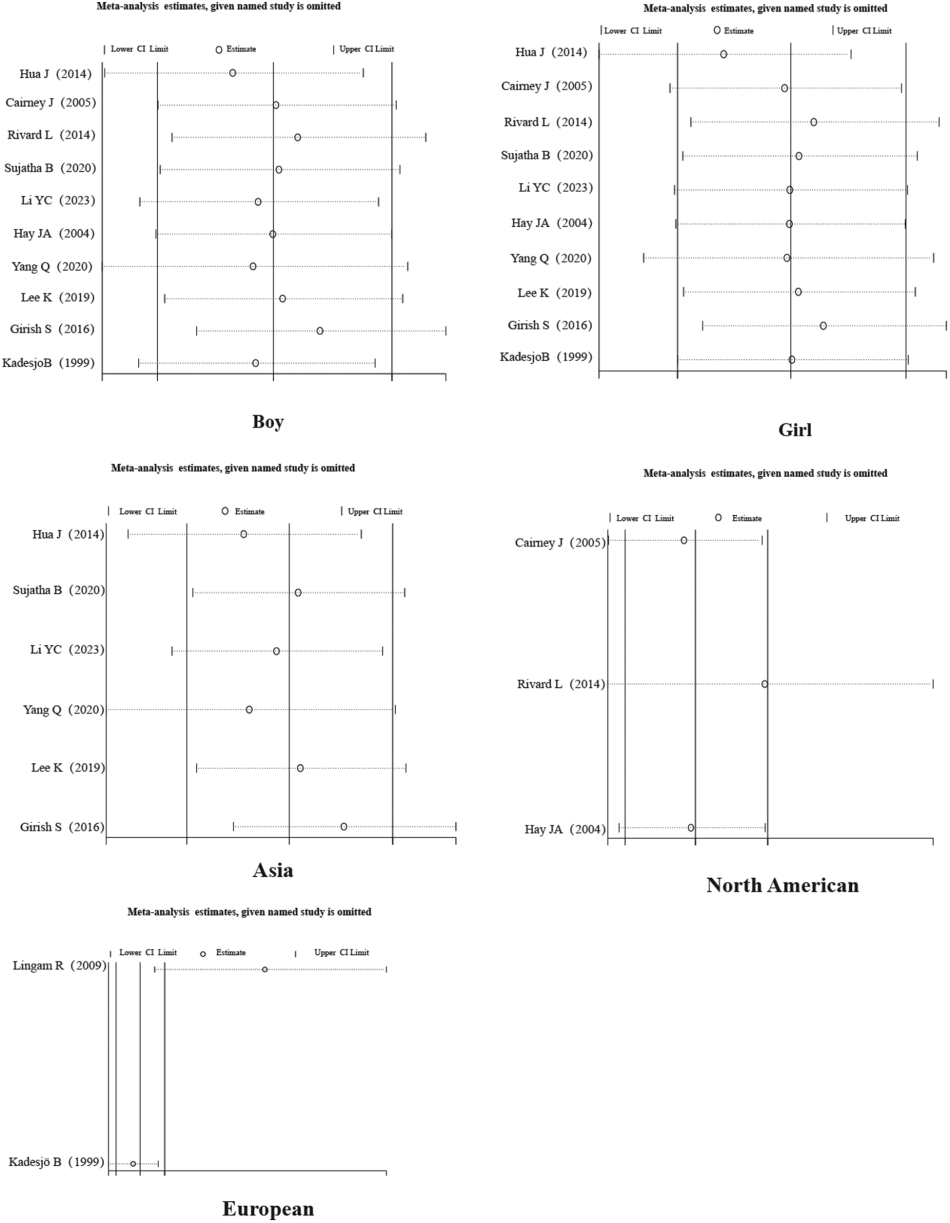
Subgroup sensitivity analysis.

**Figure 7:**
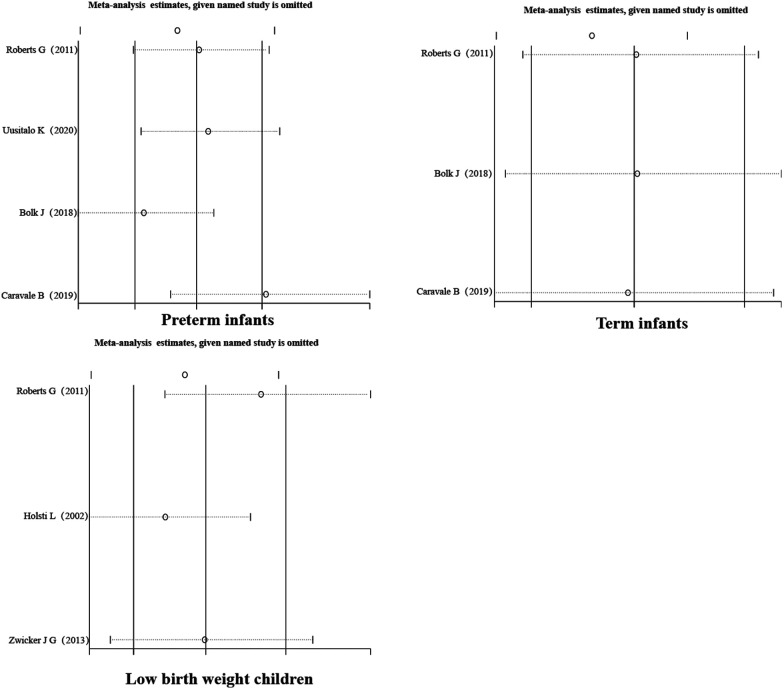
Subgroup sensitivity analysis.

A sensitivity analysis of the literature included in the meta-analysis revealed that all the literature did not differ significantly, which implies that the current study has good stability. The results are shown in Figures 5–7.

#### 3.3.4 Publication bias test

**Figure 8:**
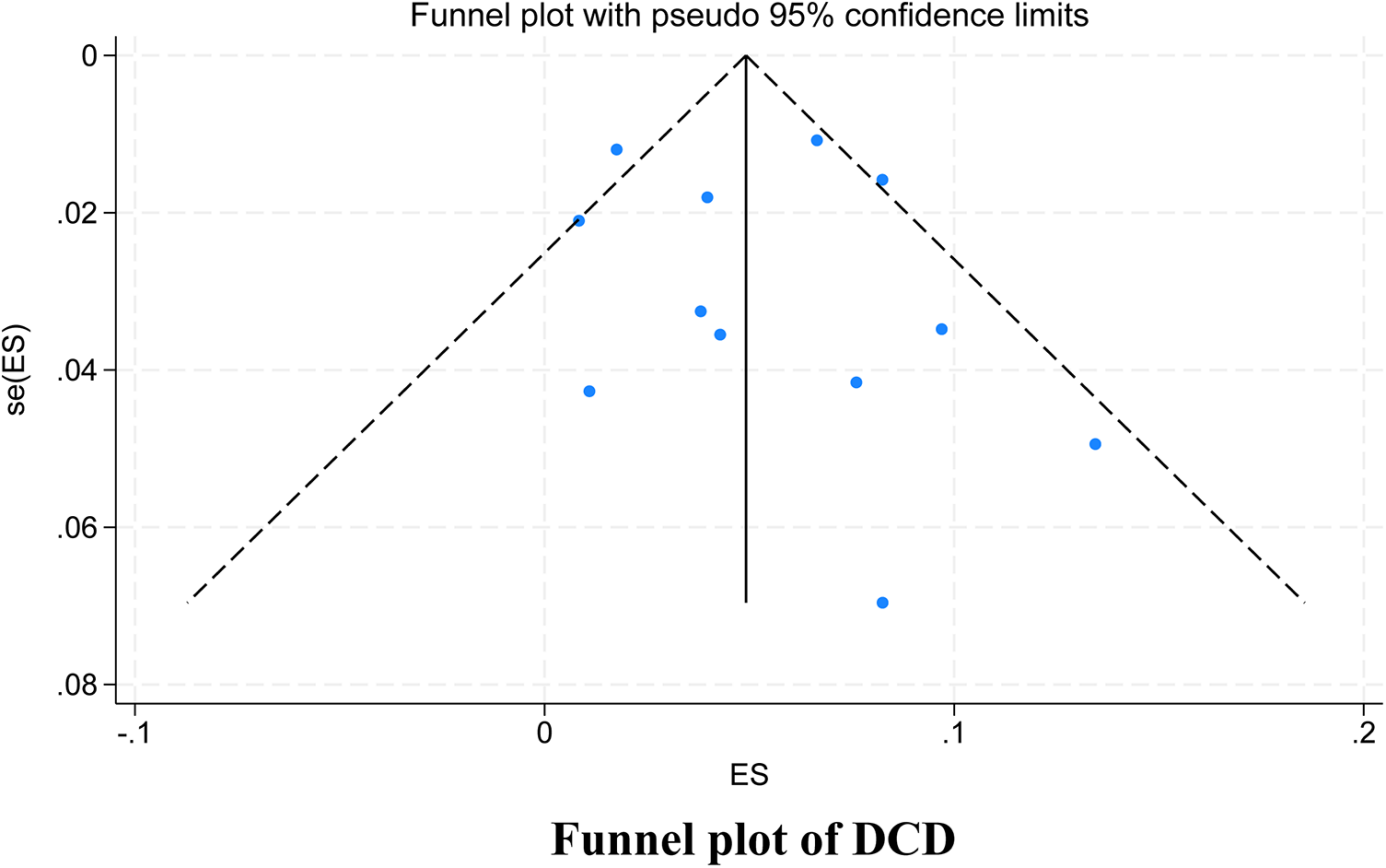
Funnel plot.

**Figure 9:**
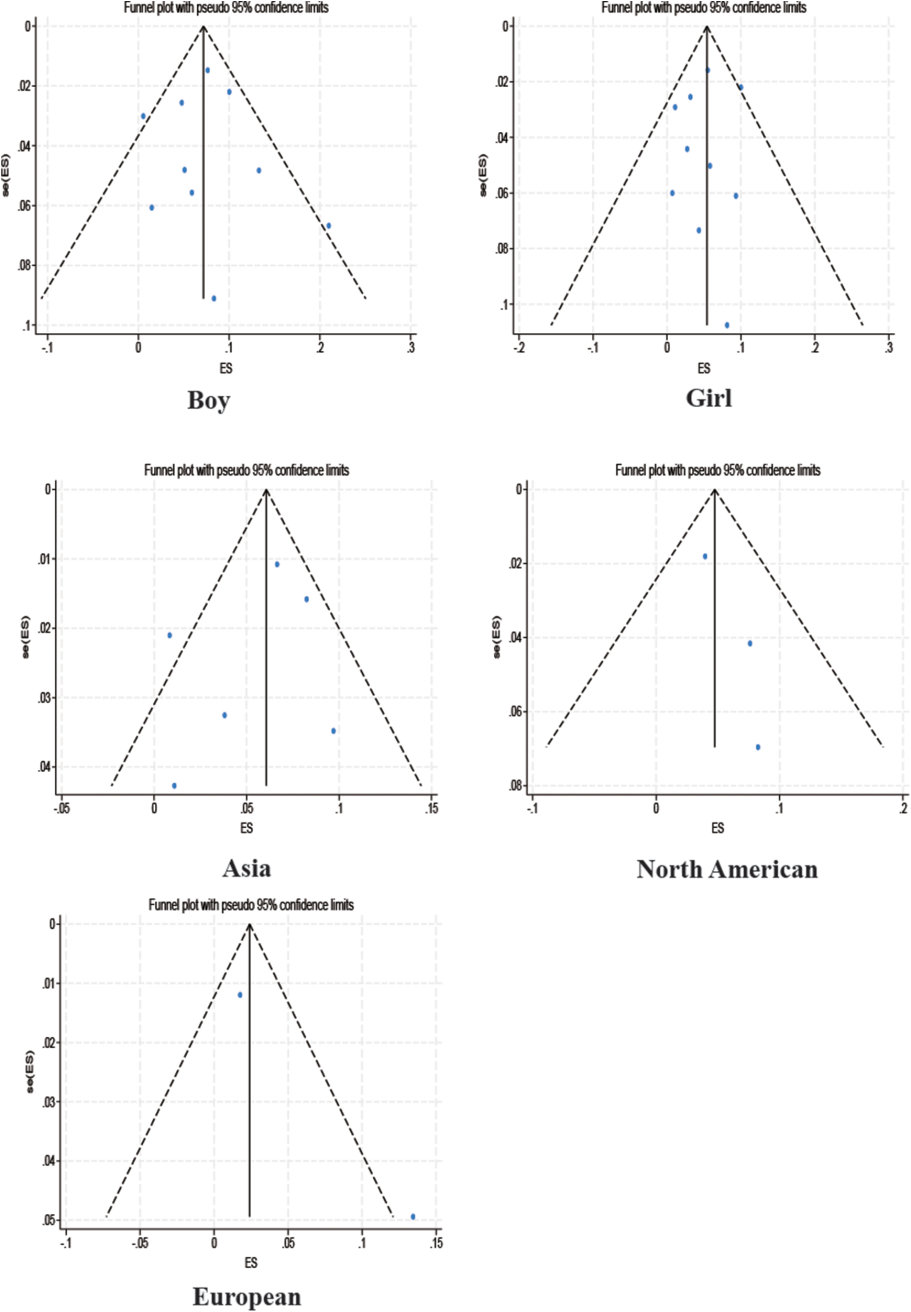
Subgroup funnel plot.

**Figure 10:**
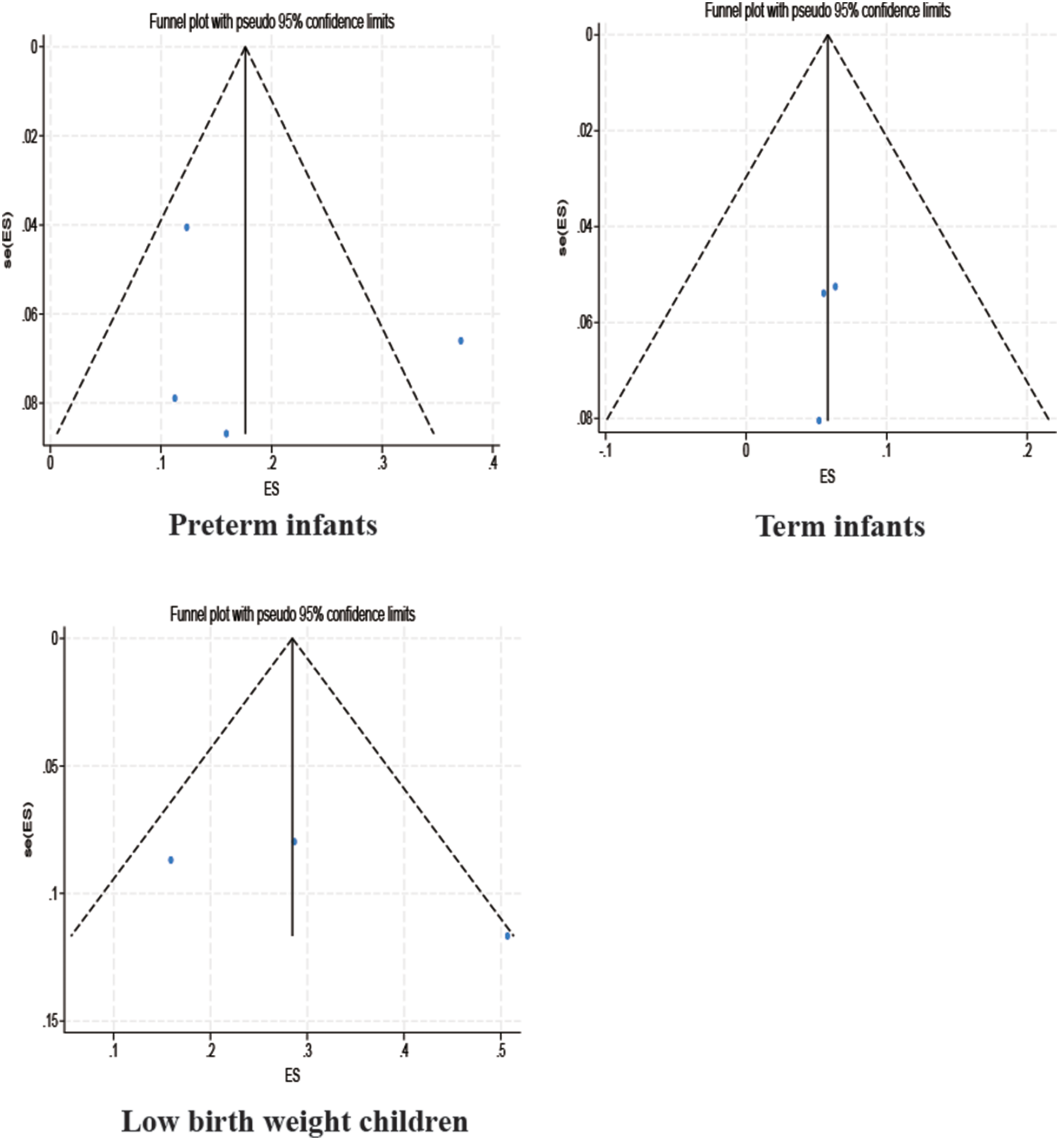
Subgroup funnel plot.

Among the studies for which the meta-analysis was performed in this study, all analyses (Begg test: *p* > 0.05; Egger test: *p* > 0.05) suggested that there was no publication bias in the study, indicating that the results of the study were relatively stable. The results are shown in Figures 8–10.

## 4 Discussion

To date, DCD is still a poorly known disease and even professional pediatricians know very little about it. Even when parents notice problems with their children and go to the hospital for consultation, they do not get an accurate diagnosis and timely intervention (36, 37). Therefore, providing an accurate prevalence of DCD and capturing the extent of disease progression is a prerequisite for conducting scientific research on DCD and drawing the attention of pediatricians and parents. Compared with previous DCD prevalence studies, our meta-analysis used a more rigorous and standardized diagnosis of DCD and was able to obtain a more accurate prevalence of DCD. In this systematic review and meta-analysis, we found that the prevalence of DCD was 5%, with a slightly higher prevalence in men than in women, consistent with the prevalence of DCD reported in the 2019 DCD Clinical Guidelines (10). The systematic evaluation by Hoorn et al. found male sex to be a risk factor for DCD, but it is noteworthy that this association was only seen in the general cohort and disappeared in the preterm cohort, with the possible reason being that the effects from preterm birth outweighed the effects of sex on DCD (38). However, in a study by Girish et al., the prevalence of DCD was found to be higher in girls than in boys (15). The relationship between sex and DCD is unclear. We suggest that this may be related to differences in brain structure between men and women, with men having a larger overall brain size than women, but women reaching peak brain volume earlier than men, and women having a smaller volume of gray matter than men but a higher density of gray matter structures, as well as differences in cerebral blood flow and thickness of cortical areas (39). Recent studies have also found that boys have a higher prevalence than girls of neurodevelopmental disorders, such as autism, attention-deficit/hyperactivity disorder, and schizophrenia, and that developmental coordination disorders, similar to these disorders, may also be affected by genetic predisposition, endocrine, and environmental factors, with sex differences (40, 41). In addition, different parenting styles and perceptions of boys and girls are also related to sex differences in motor coordination, with boys doing more outdoor activities and girls favoring fine motor activities, including beading, paper-cutting, and drawing toys (42). Prolonged performance of preferred exercise may also result in sex differences in motor function assessment.

We also found that the prevalence of DCD was significantly higher in preterm (<37 weeks) and very low birth weight children (<1,250 g) than in typical children, up to 18% and 31%, respectively. Previous studies have also found that the risk of DCD in very preterm (<32 weeks) or very low birth weight (<1,500 g) infants is six to eight times higher than that of term or typical birth weight infants, and that the risk of DCD in children born before 37 weeks is three to four times higher than that of term infants (38), which is similar to our findings. The brain develops in a specific sequence and time frame, with the total volume of gray matter in a child's brain increasing by approximately 1.4% per week starting at 29 weeks of gestation, and the total amount of white matter increasing by a factor of 5 between 35 and 41 weeks of gestation (43). The longer the gestation period, the more pronounced the regional specificity of gray matter density, which plays a key role in the establishment of effective neural networks in children, and preterm infants may be at higher risk of developing DCD due to their shorter gestational age (17). Using volumetric versus diffusion tensor imaging, Dewey et al. found that young children with DCD have smaller brain volumes in total brain tissue, cortical gray matter, cerebellum, caudate vomeronasal septum, pallidum, and thalamus, and exhibit altered white matter microstructure at 7 years of age compared with full-term births, particularly in motor areas (44). In addition, the intrauterine and extrauterine environments are different, with maternal and placental hormones playing an important role in brain development. Instead, preterm infants spend time in the neonatal intensive care unit (NICU), where different clinical courses have different effects on the shape of the brainstem and differently affect the neurodevelopmental regulatory functions of preterm infants, possibly leading to DCD (45). A study by Goyen and Lui found that DCD was independently associated with prolonged rupture of membranes in preterm infants and retinopathy of prematurity (46). The majority of VLBWI are preterm and have a higher risk of developmental delay (47). A meta-analysis performed by Pascal et al. showed that the incidence of motor developmental backwardness in VLBWI in recent years was 20.6% (48). In a sample of children with a birth weight of less than 1,500 g, Taylor et al. reported that children with a birth weight of less than 750 g had a higher risk of perceived motor difficulties than children with a birth weight of 750–1,499 g (49). Thus, most children with perceptual-motor deficits are likely to be at the low end of the birth weight range, and it is important that those birth weight groups that are most likely to have DCD should be further explored in the future.

In our study, most reports were from Asia, with a DCD prevalence of 4%. Reports from Europe were limited to two publications from two countries with a DCD prevalence of 2%, and reports from North America were limited to three publications from one country with a DCD prevalence of 6%, which was higher than that of Asia and Europe. Due to the limited number of included studies, only descriptive analyses were performed. Large-scale epidemiologic studies should be conducted in the future. Due to the insidious nature of DCD symptoms and the complexity of the diagnosis, we recommend a stepwise diagnostic strategy for children, starting with questionnaires, such as the DCD’07 questionnaire (for children aged 5–15 years) (50) or Little Developmental Coordination Disorder Questionnaire (Little DCDQ; for 3–4 year olds) screening (51). Screening questionnaires can be selected according to the age group the child is in. In addition, children with suspected DCD should be diagnosed according to the DSM5 guidelines. Special attention should be paid to premature infants and those with very low birth weights, and parents should be actively explained about the dangers of DCD and the need for early intervention. It is recommended that when learning about the basic information of the families of newly enrolled children, a column on births should be added and attached to the health records so that teachers can dynamically track the growth of the children. In recent years, professional committees for DCD diseases have been set up in medical institutions in most countries. It has been suggested that members of DCD committees actively carry out picture-based and video-based popularization of science in hospital obstetrics and gynecology departments and schools. It has also been suggested they hold public academic meetings on a regular basis, so as to raise parents’ and teachers’ awareness of DCD diseases, which will help identify children with DCD at an early stage in the family and in daycare. In addition, the study found that the writing style and handwriting of children with DCD differed significantly from normal children (52). At the primary and secondary school levels, children have a certain degree of learning ability and self-control. Therefore, schools can also widely screen children suspected of having DCD through homework writing. An example is judging the likelihood of DCD in young children based on the handwriting proficiency screening questionnaire criteria or the detailed assessment of speed of handwriting (10).

## 5 Strengths and limitations

The epidemiologic data provided by our study through a systematic review and meta-analysis are the most recent available on the prevalence of DCD worldwide. Due to the use of strict inclusion and exclusion criteria, the reports we included from the available literature provide accurate estimates that best represent the prevalence of DCD in the general pediatric population in different regions.

The study has some limitations (1). Ideally, valid estimates of DCD prevalence would require inverse probability weighting using population weights, but this was not done in this meta-analysis (2). Due to the limited number of included studies, the effects of factors such as family economic status, parental education, weight of young children, and hand habits on DCD prevalence were not further explored in the general cohort (3). The small number of included studies and the heterogeneity of the overall studies made it impossible to identify sources of heterogeneity in the literature.

## 6 Conclusion

In conclusion, our results found that the prevalence of DCD was 5%, which was stable compared to previous years but showed a higher prevalence in preterm and low birth weight children. Screening and continuous follow-up of young children for DCD is recommended, especially in preterm and low birth weight children.

## Data Availability

The original contributions presented in the study are included in the article/Supplementary Material, further inquiries can be directed to the corresponding authors.
