# Supplementary material for: The prevalence of developmental coordination disorder in children: a systematic review and meta-analysis
Source: Front Pediatr. 2024 Sep 26;12:1387406. doi: 10.3389/fped.2024.1387406 (PMC11464289; doi:10.3389/fped.2024.1387406)
Supplement: Supplementary file 1 [file Table1.pdf]

Supplementary Table 1-Literature quality assessment

NEWCASTLE - OTTAWA QUALITY ASSESSMENT SCALE COHORT STUDIES

Author	Year	Selection				Comparability	Outcome			Quality score
		Representativeness of the exposed cohort	Selection of the non exposed cohort	Ascertainment of exposure	Demonstration that outcome of interest was not present at start of study	Comparability of cohorts on the basis of the design or analysis	Assessment of outcome	Was follow-up long enough for outcomes to occur	Adequacy of follow up of cohorts	
Uusitalo K[33]	2020			★	★	★ ★	★	★		6
Caravale B[35]	2019	★	★	★	★	★ ★	★	★		8
Bolk J[30]	2018		★	★	★	★ ★	★	★		7
Zwicker J G[24]	2013	★	★	★	★	★ ★				6
Roberts G[32]	2011				★	★ ★	★	★		5
Lingam R[31]	2009	★	★	★	★	★ ★	★	★		8
Holsti L[27]	2002			★		★ ★	★	★		5

AGENCY FOR HEALTHCARE RESEARCH AND QUALITY，AHRQ

Author	Year	Define the source of information (survey, record review)	List inclusion and exclusion criteria for exposed and unexposed subjects (cases and controls) or refer to previous publications	Indicate time period used for identifying patients	Indicate whether or not subjects were consecutive if not population-based	Indicate if evaluators of subjective components of study were masked to other aspects of the status of the participants	Describe any assessments undertaken for quality assurance purposes (e.g., test/retest of primary outcome measurements)	Explain any patient exclusions from analysis	Describe how confounding was assessed and/or controlled.	If applicable, explain how missing data were handled in the analysis	Summarize patient response rates and completeness of data collection	Clarify what follow-up, if any, was expected and the percentage of patients for which incomplete data or follow-up was obtained	Quality score
Li YC[20]	2023	★	★	★	★			★			★		6
Sujatha B[28]	2023	★	★	★	★	★	★	★	★				8
Yang Q[21]	2020	★		★		★	★		★				5

Lee K[34]	2019	★	★	★	★	★	★	★	★	★			9
Girish S[15]	2016	★	★	★			★		★	★			5
Cardoso AA[29]	2014	★	★	★			★		★		★		6
Hua J[22]	2014	★	★			★	★		★		★		6
Rivard L[23]	2014	★	★	★			★		★		★		6
Cairney J[25]	2005	★	★	★			★		★		★		6
Hay J A[26]	2004	★	★	★			★		★		★		6
Kadesjö B[14]	1999	★	★	★			★		★	★	★		7
