# Supplementary material for: The prevalence of developmental coordination disorder in children: a systematic review and meta-analysis
Source: Front Pediatr. 2024 Sep 26;12:1387406. doi: 10.3389/fped.2024.1387406 (PMC11464289; doi:10.3389/fped.2024.1387406)
Supplement: Supplementary file 2 [file Datasheet1.pdf]

Pubmed search:

Step	Search formula
#1	"Incidence"[Mesh]
#2	Morbidit*[Title/Abstract] OR Prevalence*[Title/Abstract] OR epidemi*[Title/Abstract] OR incidence* [Title/Abstract] OR frequenc*[Title/Abstract] OR occurrence*[Title/Abstract]
#3	#1OR#2
#4	“Motor Skills Disorders"[Mesh]
#5	Developmental Coordination Disorder[Title/Abstract] OR Coordination Disorder, Developmental[Title/Abstract] OR Developmental Coordination Disorders[Title/Abstract] OR Clumsiness[Title/Abstract] OR Clumsy [Title/Abstract] OR Clumsy child syndrome[Title/Abstract] OR Clumsy child [Title/Abstract] OR In-coordination[Title/Abstract] OR Dys-coordination[Title/Abstract] OR Minimal brain dysfunction[Title/Abstract] OR Minor neurological dysfunction/disorder[Title/Abstract] OR Motor delay[Title/Abstract] OR Perceptual-motor impairment[Title/Abstract] OR Motor coordination difficulties /problems[Title/Abstract] OR Motor learning difficulties /problems[Title/Abstract] OR Mild motor problems[Title/Abstract] OR Non-verbal learning disability /disorder/dysfunction[Title/Abstract] OR Sensorimotor difficulties[Title/Abstract] OR Sensory integrative dysfunction[Title/Abstract] OR Physical awkwardness[Title/Abstract] OR Physically awkward[Title/Abstract] OR Psychomotor disorders[Title/Abstract] OR Apraxias[Title/Abstract] OR Developmental dyspraxia[Title/Abstract] OR Perceptual motor dysfunction[Title/Abstract] OR Minimal cerebral dysfunction[Title/Abstract]
#6	#4OR#5
#7	"Child"[Mesh]
#8	Children[Title/Abstract] OR childhood[Title/Abstract] OR youth[Title/Abstract] OR adolescent[Title/Abstract] OR adolescence[Title/Abstract]
#9	#7OR#8
#16	#3AND#6AND#9

Embase search:

Step	Search formula
#1	'Incidence':ab,ti OR 'Morbidit*':ab,ti OR 'Prevalence*':ab,ti OR 'epidemi*':ab,ti OR 'incidence*':ab,ti OR 'frequenc*':ab,ti OR

	'occurrence*':ab,ti
#2	'Motor Skills Disorders':ab,ti OR 'Developmental Coordination Disorder':ab,ti OR 'Coordination Disorder, Developmental':ab,ti OR 'Clumsiness':ab,ti OR 'Clumsy':ab,ti OR 'Clumsy child syndrome':ab,ti OR 'Clumsy child':ab,ti OR 'In-coordination':ab,ti OR 'Minimal brain dysfunction':ab,ti OR 'Minor neurological dysfunction/disorder':ab,ti OR 'Motor delay':ab,ti OR 'Perceptual-motor impairment':ab,ti OR 'Motor coordination difficulties /problems':ab,ti OR 'Motor learning difficulties /problems':ab,ti OR 'Mild motor problems':ab,ti OR 'Non-verbal learning disability /disorder/dysfunction':ab,ti OR 'Sensorimotor difficulties':ab,ti OR 'Sensory integrative dysfunction':ab,ti OR 'Physical awkwardness':ab,ti OR 'Physically awkward':ab,ti OR 'Psychomotor disorders':ab,ti OR 'Apraxias':ab,ti OR 'Developmental dyspraxia':ab,ti OR 'Perceptual motor dysfunction':ab,ti OR 'Minimal cerebral dysfunction':ab,ti
#3	'Child':ab,ti OR 'Children':ab,ti OR 'childhood':ab,ti OR 'youth':ab,ti OR 'adolescent':ab,ti OR 'adolescence':ab,ti
#4	#1 AND #2 AND #3AND#4

Web of science search:

Step	Search formula
#1	TS=(Incidence OR Morbidit* OR Prevalence* OR epidemi* OR incidence* OR frequenc* OR occurrence*)
#2	TS=(Motor Skills Disorders OR Developmental Coordination Disorder OR Coordination Disorder, Developmental OR Developmental Coordination Disorders OR Clumsiness OR Clumsy OR Clumsy child syndrome OR Clumsy child OR In-coordination OR Dys-coordination OR Minimal brain dysfunction OR Minor neurological dysfunction/disorder OR Motor delay OR Perceptual-motor impairment OR Motor coordination difficulties ∕ problems OR Motor learning difficulties ∕problems OR Mild motor problems OR Non-verbal learning disability ∕disorder∕dysfunction OR Sensorimotor difficulties OR Sensory integrative dysfunction OR Physical awkwardness OR Physically awkward OR Psychomotor disorders OR Apraxias OR Developmental dyspraxia OR Perceptual motor dysfunction

	OR Minimal cerebral dysfunction)
#3	TS=(Child OR Children OR childhood OR youth OR adolescent OR adolescence）
#6	#1 AND #2 AND #3

The Cochrane Library search :

Step	Search formula
#1	"Incidence"
#2	(Morbidit*):ab,ti,kw OR (Prevalence*):ab,ti,kw OR (epidemi*):ab,ti,kw OR (incidence*):ab,ti,kw OR (frequenc*):ab,ti,kw OR (occurrence*):ab,ti,kw
#3	#1OR#2
#4	"Motor Skills Disorders"[Mesh]
#5	(Developmental Coordination Disorder):ab,ti,kw OR (Coordination Disorder, Developmental):ab,ti,kw OR (Developmental Coordination Disorders):ab,ti,kw OR (Clumsiness):ab,ti,kw OR (Clumsy):ab,ti,kw OR (Clumsy child syndrome):ab,ti,kw OR (Clumsy child):ab,ti,kw OR (In coordination):ab,ti,kw OR (Dysplasia coordination):ab,ti,kw OR (Minimal brain dysfunction):ab,ti,kw OR (Minor neurological dysfunction / disorder):ab,ti,kw OR (Motor delay):ab,ti,kw OR (Perceptual motor impairment):ab,ti,kw OR (Motor coordination difficulties / problems):ab,ti,kw OR (Motor learning difficulties / problems):ab,ti,kw OR (Mild motor problems):ab,ti,kw OR (None verbal learning disability / disorder/dysfunction):ab,ti,kw OR (Sensorimotor difficulties):ab,ti,kw OR (Sensory integrative dysfunction):ab,ti,kw OR (Physical awkwardness):ab,ti,kw OR (Physically awkward):ab,ti,kw OR (Psychomotor disorders):ab,ti,kw OR (Apraxias):ab,ti,kw OR (Developmental dyspraxia):ab,ti,kw OR (Perceptual motor dysfunction):ab,ti,kw OR (Minimal cerebral dysfunction):ab,ti,kw
#6	#4OR#5
#7	"Child"
#8	(Children):ab,ti,kw OR (childhood):ab,ti,kw OR (youth):ab,ti,kw OR (adolescent):ab,ti,kw OR (adolescence):ab,ti,kw
#9	#7OR#8
#10	#3AND#6AND#9

CINAHL search

Step	Search formula
#1	'Incidence' OR 'Morbidit*' OR 'Prevalence*' OR 'epidemi*' OR 'incidence*' OR 'frequenc*' OR 'occurrence*'
#2	'Developmental Coordination Disorder' OR 'Coordination Disorder, Developmental' OR 'Developmental Coordination Disorders' OR 'Clumsiness' OR 'Clumsy' OR 'Clumsy child syndrome' OR 'Clumsy child' OR 'In-coordination' OR 'Dys-coordination' OR 'Minimal brain dysfunction' OR 'Minor neurological dysfunction/disorder' OR 'Motor delay' OR 'Perceptual-motor impairment' OR 'Motor coordination difficulties ⁄ problems' OR 'Motor learning difficulties ⁄problems' OR 'Mild motor problems' OR 'Non-verbal learning disability ⁄disorder⁄dysfunction' OR 'Sensorimotor difficulties' OR 'Sensory integrative dysfunction' OR 'Physical awkwardness' OR 'Physically awkward' OR 'Psychomotor disorders' OR 'Apraxias' OR 'Developmental dyspraxia' OR 'Perceptual motor dysfunction' OR 'Minimal cerebral dysfunction'
#3	'Child' OR 'Children' OR 'childhood' OR 'youth' OR 'adolescent' OR 'adolescence'
#4	#1 AND #2 AND #3

PsycINFO

Step	Search formula
#1	'Incidence' OR 'Morbidit*' OR 'Prevalence*' OR 'epidemi*' OR 'incidence*' OR 'frequenc*' OR 'occurrence*'
#2	'Developmental Coordination Disorder' OR 'Coordination Disorder, Developmental' OR 'Developmental Coordination Disorders' OR 'Clumsiness' OR 'Clumsy' OR 'Clumsy child syndrome' OR 'Clumsy child' OR 'In-coordination' OR 'Dys-coordination' OR 'Minimal brain dysfunction' OR 'Minor neurological dysfunction/disorder' OR 'Motor delay' OR 'Perceptual-motor impairment' OR 'Motor coordination difficulties ⁄ problems' OR 'Motor learning difficulties ⁄problems' OR 'Mild motor problems' OR 'Non-verbal learning disability ⁄disorder⁄dysfunction' OR 'Sensorimotor difficulties' OR 'Sensory integrative dysfunction' OR

	'Physical awkwardness' OR 'Physically awkward' OR 'Psychomotor disorders' OR 'Apraxias' OR 'Developmental dyspraxia' OR 'Perceptual motor dysfunction' OR 'Minimal cerebral dysfunction'
#3	'Child' OR 'Children' OR 'childhood' OR 'youth' OR 'adolescent' OR 'adolescence'
#4	#1 AND #2 AND #3
